# Validating the use of pen scores to capture behaviors expressed by cattle unrestrained in a pen

**DOI:** 10.1093/jas/skaf026

**Published:** 2025-02-05

**Authors:** Jamie T Parham, Jessica J Schmidt, Amy E Tanner, Mark L Wahlberg, Temple Grandin, Ronald M Lewis

**Affiliations:** Division of Animal Sciences, University of Missouri, Columbia, MO 65211, USA; Department of Animal Science, University of Nebraska - Lincoln, Lincoln, NE 68583, USA; Department of Animal Science, University of Nebraska - Lincoln, Lincoln, NE 68583, USA; Department of Animal and Poultry Sciences, Virginia Tech, Blacksburg, VA 24061, USA; Department of Animal and Poultry Sciences, Virginia Tech, Blacksburg, VA 24061, USA; Department of Animal Sciences, Colorado State University, Fort Collins, CO 80521, USA; Department of Animal Science, University of Nebraska - Lincoln, Lincoln, NE 68583, USA

**Keywords:** beef cattle, group pen score, individual pen score, nonrestrained behavior, temperament, validating ethograms

## Abstract

Many subjective methods exist to assist producers in evaluating temperament. Use of a pen test, which allows behavior to be observed in a nonrestrained setting, may be an effective method to evaluate temperament by allowing more variation among animals to be expressed as compared to a restrained test. The objective of this study was to analyze video recordings of penned calves to determine whether the repertoire of behaviors they expressed was adequately captured by their individual pen score (**IPS**) and group pen score (**GPS**). In each of 3 yr, 40 predominantly Angus commercial *Bos taurus* heifers, 2-wk postweaning , were assessed monthly in 3 consecutive months (recording periods). After routine handling through a weigh crate and squeeze chute, each heifer was released individually into a pen (12 × 6 m). Their response to 30 s of human presence within the pen was evaluated. They were then joined in groups of four in a larger (12 × 8 m) pen and re-evaluated for 30 s in a group setting. An IPS and GPS were assigned using an ethogram, with scores ranging from 1 (docile) to 6 (aggressive). This interaction was also video recorded. Using the videos, the explicit behaviors captured by each pen score were investigated using Noldus Observer software. Data were analyzed with ANOVA using SAS. Within each recording period, the concordance of pen scores with the repertoire of behaviors expressed was compared. In the group setting, heifers could not be clearly distinguished on video and were instead assigned the average GPS of the group. Heifers with higher IPS and average GPS categories had larger flight zone sizes and reached faster paces for longer periods of time. Furthermore, heifers with lower IPS appeared more neutral to the presence of a human and moved around less often. Ethograms for pen score successfully delineated the temperament of heifers. The use of such scores can provide a valuable on-farm assessment of cattle behavior during routine handling.

## Teaser

Removing restraint allows for a more comprehensive evaluation of the temperament of an animal. When explicit behaviors described within the pen score ethograms were categorized and quantified, they closely aligned with a designated score, supporting the conclusion that pen score is a valuable and relevant summary of the repertoire of behaviors associated with temperament in cattle.

## Introduction

Temperament is an animal’s behavioral response in a single context, measured on some biological scale (Mackay and Haskell, 2015). To effectively select for docility in cattle, the criteria measured should be indicative of behavior during normal handling practices (Fordyce et al., 1982). Commonly, temperaments are quantified based on behaviors when cattle are restrained in a squeeze chute [chute score (**CS**) (Tulloh, 1961)] and when exiting from the squeeze chute [exit score (**ES**) (BIF Guidelines, 2010) and exit velocity (**EV**) (Burrow et al., 1988)]. Another, although less common, evaluation of temperament is conducted when an animal is isolated in a pen without restraint. Such pen scores (King et al., 2006) can be assessed on animals individually and in small groups. Like CS and ES, they provide attractive indicators of behavior because of their ease of use on-farm.

This study is the culmination of a series of investigations that both developed and validated behavioral measurements associated with stress during handling in beef heifers at weaning. First, using measures of interclass correlation, we determined that neither CS nor ES was susceptible to the previous experience of the individual (observer) who scored the measure (Parham et al., 2019a). Second, we investigated the relationships among CS, ES, EV, individual pen score (**IPS**), and group pen score (**GPS**), and whether stress changed under repeated, routine management practices (Parham et al., 2019b, 2022). We concluded that excitable cattle acclimated to repeated, calm handling in the chute and individual pen. Our third objective was to determine if subjective measures like CS and ES indicated true physiological stress during handling (Parham et al., 2021). Although levels of metabolites did not significantly change with repeated handling, our subjective measures of behavior around the chute provided a reasonable indication of physiological stress.

Our subjective evaluations of temperament involved use of ethograms that combined several behaviors into a single score. That approach has the clear benefit of its ease in implementation. However, it could also have drawbacks. For example, IPS blends the animal’s pace or speed, flight zone, head placement, and location in the pen. It is unlikely that the behaviors expressed by animals perfectly align with the description assigned to an individual numerical score. Such variability could lead to misclassification of animals’ IPS depending on an observer’s prior experience. For instance, consider flight zone. It is the distance at which a person can approach an animal before it moves away. Cattle in close daily proximity to people become acclimated and have smaller flight zones than genetically similar cattle raised on pasture (Grandin and Deesing, 2022) simply due to the environment in which they are raised. Such variation in flight zone sizes may, therefore, impact the stress response animals portray when they are secluded in a pen with a human. This, in turn, may affect the behaviors they express. For example, an animal’s attempts to escape from the perceived threat may affect the rate and direction of their movement within a pen, perhaps resulting in their colliding into a barrier (fence). For pen scores to be effective, they must sufficiently and consistently differentiate cattle temperament despite such nuances in the expressions of behaviors.

The objective of this final study, therefore, was to analyze video recordings of calves penned individually and in groups with a stressor (human) and determine whether their IPS and GPS, respectively, adequately capture the repertoire of behaviors they expressed in each setting. Since the ethograms were designed to consider the likely behaviors that calves would express in these situations, our hypothesis was that the scores would sufficiently portray their behaviors. Still, before an ethogram is applied generally, its efficacy needs to be thoroughly validated. Not only can the results of this study be used to address the challenges of concatenating a multitude of behaviors into a single subjective score, but also to better understand the benefits and limitations of evaluating temperament when animals are penned individually or in a group.

## Materials and Methods

All procedures and protocols used in this study were approved by the Institutional Animal Care and Use Committee at Virginia Tech.

### Experimental animals

This study builds on previous work evaluating temperament in heifers shortly after weaning when confined in a squeeze chute (Parham et al. 2019a, 2019b, 2021, 2022). Across 3 consecutive years, 120 commercial *Bos taurus* (75% Angus or more) spring-born heifer calves (*n* = 40 per year) were reared at the Virginia Tech Shenandoah Valley Agricultural Research and Extension Center in Steeles Tavern, Virginia, United States, with their respective dams until weaning (185 ± 11 d in age). The heifers were daughters of 21 sires, ranging from 1 to 23 daughters per sire.

Calves were exposed to working facilities on two separate occasions up through weaning: at approximately 2½ mo in association with breeding of their dams, and at approximately 6 mo when weaned. At each event, they were vaccinated and dewormed. The facilities included a series of holding pens. An adjoining alleyway led to a curved alley, which then led to a weigh crate and separate squeeze chute (Pearson Manual Chute).

Once separated from their dams at weaning, the calves completed a 1-wk fence line weaning period. The heifers were then transported to Virginia Tech Kentland farm, Virginia, United States. They were placed in a single management group on grass for approximately 1 mo prior to the start of the current study.

### Experimental design and data collection

In each of 3 yr, data were collected across three recording periods, each 1 mo apart (i.e., recording period 1 [October], period 2 [November], and period 3 [December]) starting on the second Monday or Tuesday of October and repeated at 4-wk intervals. During each recording period, all 40 heifers were moved into a holding pen. Four heifers were then randomly drawn from the group and calmly herded into the cattle-handling facility. The facility consisted of a small holding pen narrowing into a curved alley that led to a weigh crate and separate squeeze chute. One at a time, heifers were weighed and moved into the squeeze chute (Priefert Model S04) where their head was secured in the head gate and the sides of the chute left opened with no restriction on the body. The bottom of the chute was wide enough that heifers were easily in a standing position.

On each observation day during the study, three experienced observers simultaneously recorded the heifer’s CS (Tulloh, 1961) within the first 15 s of being placed in the squeeze chute. Heart rate, rectal temperature, and a fecal and jugular blood sample were then taken. Upon release from the chute, an ES (BIF Guidelines, 2010) was recorded by the same individuals. A flight speed (s) also was measured using electronic timers (Polaris, FarkTek, Wylie, TX) over a 2 m distance, based on the principle developed by Burrow et al. (1988), beginning 1 m from the head of the chute. This value was converted into an EV (m/s) for analysis, where larger values corresponded with more excitable heifers.

Following this routine handling, each heifer was calmly walked to a 12 × 6 m pen. Although fencing was open-sided, there was no direct or visual, and limited hearing, contact with animals in other areas of the working facility. Once in the individual pen, heifers were exposed to the same human stressor across all observation days. This person wore insulated clothing of a similar style to the other study participants taking measurements and did their best to wear the exact same clothing articles during each cattle handling experience. After closing the gate, this person walked in and stood in the center of the pen for 30 s. Although they stood in the same location, they rotated to always be facing the heifer as she moved about the pen. During this time, IPS were assigned independently by the same experienced observers using an ethogram based on King et al. (2006) (Table 1). If a heifer was deemed too excitable by the human stressor (e.g., running around the pen, attempting to escape), they did not enter but stood just outside the pen yet contiguous to its center. In such cases, the heifer still received a representative IPS.

**Table 1.** Pen score ethogram used to measure temperament in heifers both individually and in a group

Pen score	IPS description^1^	GPS description^1^
1. Docile	Walks slowly, can be approached slowly, not excited by humans.	Walks slowly, can be approached slowly, not excited by humans.
2. Slightly Restless	Aware of humans, head up, moves away from approaching human, runs fence line, stops and looks around.	Aware of humans, head up, moves slowly away from approaching human.
3. Restless	Constantly runs along fence line, head up.	Runs along fences, stands in corner if humans stay away.
4. Nervous	Agitated, runs along fence line, head up, looking for a way of escape, and will run if humans come closer, stops before hitting gates and fences, avoids humans.	Runs along fences, head up and will run if humans come closer, stops before hitting gates and fences, avoids humans.
5. Very Nervous	Runs, head high and very aware of humans, may run into fences and gates, flighty.	Runs, stays in back of the group, head high and very aware of humans, may run into fences and gates.
6. Wild (Aggressive)	Excited, runs into fences, runs over anything in its path.	Excited, runs into fences, runs over anything in its path.

^1^Adapted from King et al. (2006).

Each heifer was then moved into a 12 × 8 m pen, also with open siding. Pens were separated by an alleyway with no direct and limited visual or hearing contact between them. Heifers remained in the group pen until four heifers had been moved through the working facilities and placed together. Once in a group of four, a similar ethogram [adapted from King et al. (2006); Table 1] was used to assign a GPS to each heifer individually by the same observers, based on their reaction to the same human stressor. In this case, the person walked to the center of the pen, paused, and then continued diagonally in the direction of the group of heifers once before returning to the center of the pen. This process was repeated until all 40 heifers had been observed.

### Video analyses

Continuous focal sampling was conducted by one experienced person who viewed the individual and group pen score video recordings of each heifer, or group of heifers, using Observer software (Noldus, the Netherlands). Descriptions of the behaviors evaluated on an individual or a group are provided in Tables 2 and 3, respectively. Individual heifers could not be clearly distinguished in some group pen videos. However, the mean GPS for all heifers from each of the recording periods [1.72 ± 0.05, 1.58 ± 0.04, and 1.51 ± 0.04, respectively, for recording periods 1, 2, and 3 (Parham et al., 2022)] provided a reasonable statistic to capture the overall behavior of groups. Therefore, evaluation of heifers gathered in the pen was related back to the behavior of the group as a whole instead of each individual animal. Since the individual who evaluated the videos was not an observer present during the study, this individual will be referred to as the viewer.

**Table 2.** Description of detailed behaviors used to evaluate individual pen score videos

Type	Ethogram	Description
Categorical	Human entry	1. Stays for duration: human feels it is safe to remain in pen with animal for duration of observation. 2. Enters but does not stay for duration: human feels it is safe to enter pen with animal but feels unable to remain for duration of observation. 3. Never enters pen: human does not feel safe to enter into pen with animal.
	Fight or flight	1. Neutral: heifer not disturbed by human presence. May move around pen. 2. Attempt to escape (flight): heifer’s eyes oriented towards human, moves away from threat. May look for a way of escape by sticking head out of or underneath fence. 3. Attack (fight): heifer is threatened to a point where she pursues the human standing inside or outside of the pen.
	Flight zone size	1. Low: heifer stands still, minimal movement. Human is clearly not within the heifer’s flight zone. 2. Medium: human may be in heifer’s flight zone, or on the perimeter of it. Heifer clearly reacts to the presence of the human but responds in a temperate manner (mildly or moderately) to distance herself from human. Not as reactive as High. 3. High,: (given this score automatically if human does not enter pen) human is clearly in the heifer’s flight zone, which may be larger than the pen. Wildly moves about pen. May charge human.
Counts	Counted behaviors	1. Number of threats (attempt to charge but turns away in different direction). 2. Number of times head-butts fence (actively or coincidentally due to sliding). 3. Number of times hits fence (hip or shoulder check). 4. Number of times attempts to escape (puts head through gate, etc.). 5. Number of tail movements (quantified as a deviation from vertical).
Timed	Pace	1. Stationary: no movement. 2. Fidgets: small movements back and forth through nervousness. 3. Walk: four-time movement. Each leg moves on its own and in a set order. 4. Trot: two-time movement. Two diagonal pairs of legs in motion at once. 5. Run/canter: The canter is a three-time rhythm. There is either left lead or right lead canter. The run is a four-beat rhythm. All four feet are never on the ground at the same time. The run progresses out of canter. 6. Out of view of the camera.
	Direction of movement	1. Backwards/turns around: heifer changes direction of movement. 2. Down single side of pen, stops: heifer moves along the fence but is able to stop before coming to the corner. 3. Down single side of pen, slides: heifer moves along the fence with such momentum that she slides (feet skid) in the gravel. May run into the fence. 4. Circles the pen: heifer continuously moves about the pen in a circular or erratic pattern. May be half or full circles. 5. Travels through pen, diagonally: (occurs most often when human is not in pen) heifer starts in one corner of pen and travels through the middle instead of going down the side.

**Table 3.** Description of detailed behaviors used to evaluate group pen score videos

Type	Ethogram	Description
Categorical	Group flight zone size	1. Low: group of heifers stand still, minimal movement. Human is clearly not within their flight zone until much later in approach. 2. Medium: human may be in the group’s flight zone, or on the perimeter of it, during approach. Group clearly reacts to the presence of the human, but responds in a temperate manner (mildly or moderately) to distance themselves from human. Not as reactive as High. 3. High: human is clearly in the group’s flight zone upon initial approach, which may be larger than the pen. Wildly move about pen. May charge human.
Timed	(Average) Pace	1. Stationary: no movement. 2. Fidgets: small movements back and forth through nervousness. 3. Walk: four-time movement. Each leg moves on its own and in a set order. 4. Trot: two-time movement. Two diagonal pairs of legs in motion at the same time. 5. Run/canter: The canter is a three-time rhythm. There is either left lead or right lead canter. The run is a four-beat rhythm. All four feet are never on the ground at the same time. The run progresses out of canter. 6. Out of view of camera.
	Willingness to separate	1. Together: all heifers in the group travel/are stationary together. 2. Separate but rejoin: one or more of the heifers are split from the group, with immediate attempt (<2 s) to rejoin. 3. Separate: One or more of the heifers are split from the group (greater than one cow length), with no movement to rejoin.

#### Video analysis of individual pen

**Figure 1.**
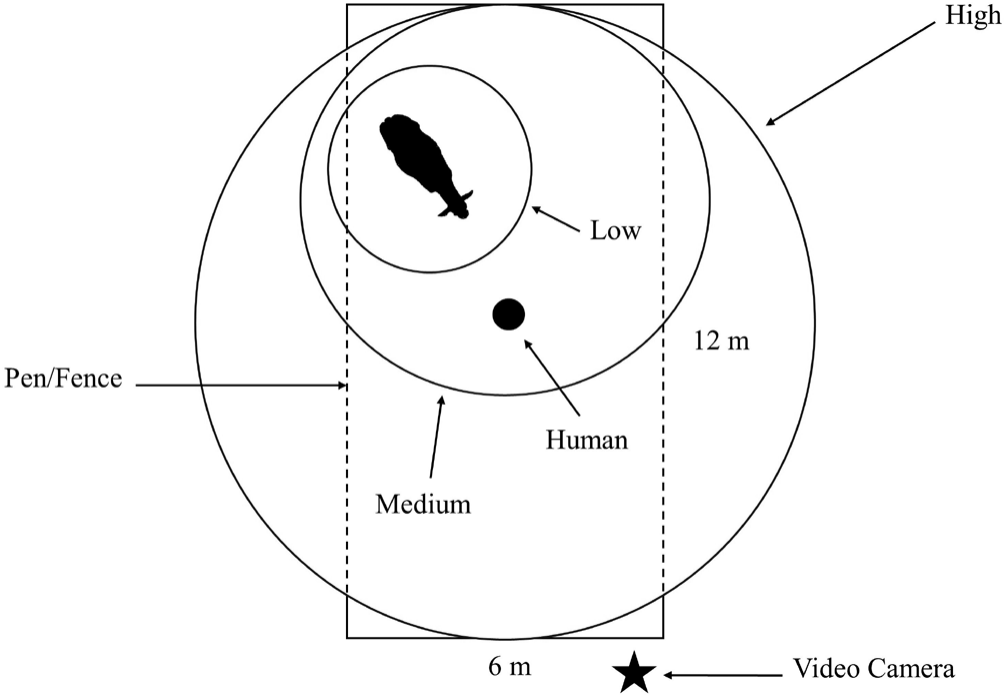
Diagram of the individual pen environment depicting flight zone sizes of low, medium, or high.

The viewer recorded several categorical behaviors based on the entire video (Table 2). First was whether the human felt safe to enter the pen with the heifer, or entered the pen but did not stay for the duration of the test. The viewer also evaluated the reaction of the heifer to the human as initiating flight or fight instincts, or if the heifer appeared neutral to the individual’s presence. Similarly, the viewer recorded a flight zone size as either low, medium, or high, as shown in Figure 1. Heifers that received a low flight zone size were more comfortable standing still in the presence of a human, with minimal movement. In this case, the human was clearly not encroaching on their flight zone. A medium flight zone was assigned when the heifer reacted to the human presence, but in a more moderate manner. Typically, the heifer would move away from the human stressor towards the corners or sides of the pen, with the heifer’s flight zone necessarily no larger than the confines of the pen. Lastly, a heifer received a high flight zone size if her reaction to the human was excitable, resulting in rapid, wild, and incessant movement about the pen. In this case, it was assumed the flight zone was larger than the pen itself. If the human did not feel safe entering the pen with a heifer, she automatically received a high flight zone size and a reaction of “fight” instincts.

As a count variable, the viewer recorded the number of times each heifer threatened or charged the human, and the number of times she head-butted the fence (either actively or coincidently), hit the fence, attempted to escape, or flicked her tail (Table 2).

**Figure 2.**
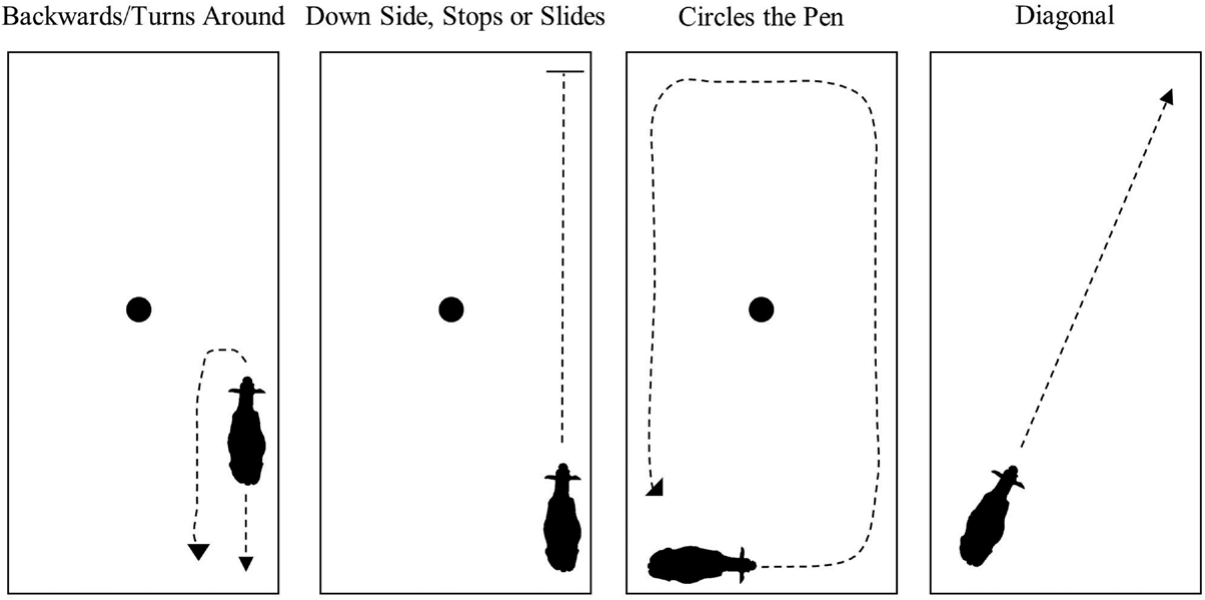
Diagram of the individual pen environment depicting direction of movement behaviors.

Lastly, several timed activities were recorded if their duration was longer than one second (Table 2). These activities were the pace of the animal (stationary, fidgets, walks, trots, runs/canters) and their direction of movement (Figure 2). From this information, the fastest pace reached by each heifer was recorded. Videos ranged in length from 25 to 32 s. Therefore, timed activities were standardized to ensure consistent comparisons. This was done by dividing each separate timed activity by the total time of the video, then multiplying that by 30 s.

#### Video analysis of group pen

To compare group behavior to individual responses, the group pen scores for each heifer in each group were averaged to obtain an overall score. As a categorized behavior (Table 3), the flight zone size of the group was assigned at the initial approach of the human based on the reaction of a majority of the group. In the rare instance when there was no majority, the higher of the flight zone sizes were recorded.

The amount of time spent moving was also recorded for each animal for each pace classification (Table 3). Because these evaluations were summarized on a group basis, time spent by each heifer for a pace was averaged across the group. Like the analysis of heifers when individually penned, the fastest pace reached by the group was also recorded. The other timed activity for the group included the duration heifers were willing to remain separate from one another (visual distance greater than one cow length). All timed activities were again standardized to 30 s.

### Statistical analyses

Interobserver reliability, or the consistency among the three observers, of both IPS and GPS was calculated using Fleiss’ kappa coefficient (*K*) and an intraclass correlation coefficient (**ICC**). All reliability calculations were carried out using the irr package (Gamer et al., 2020) in R.

The IPS and GPS recorded on each cow by the three observers were averaged within an observation day to obtain a representative score. Pearson correlations were calculated between IPS and GPS for all heifers for each recording period separately in R (R Core Team, 2013). In all cases, the correlation coefficients did not differ in magnitude between recording events (*P* > 0.10). Therefore, the Pearson correlations were estimated for the combined data. The value of the combined coefficient was tested for whether it was significantly different from zero (*P* < 0.05).

Heifers were then categorized within IPS category. This was based on their average IPS on a given day being less than 2.0 (*n* = 178), equal to 2.0 but less than 3.0 (*n* = 77), equal to 3.0 but less than 4.0 (*n* = 39), equal to 4.0 but less than 5.0 (*n* = 19), and greater than or equal to 5.0 (*n* = 11). The GPS were much lower on average, with no average GPS being greater than 4.0. Cows, therefore, were categorized based on their average GPS being less than 2.0 (*n* = 60), equal to 2.0 but less than 3.0 (*n* = 19), and 3.0 or greater (*n* = 7).

#### Video analyses of categorical behaviors

Several of the behaviors associated with the individual and group pen evaluations were categorical in their measure: flight zone size (low, medium, high), fastest pace (stationary, fidget, walk, trot, canter/run), fight or flight (neutral, fight, flight), and whether the observer felt safe to enter the pen (yes or no). Since observations in categories were counted, these data were analyzed using ordered contingency tables as well as log-linear models. Contingency tables and *P*-values were obtained using the “coin” (Hothorn et al., 2008) and “rcompanion” (Mangiafico, 2020) packages in R.

#### Video analyses of counted behaviors

When heifers were individually penned, several behaviors collected were counts. In such cases, these observations were analyzed using the GLIMMIX procedure in SAS (SAS Institute Inc., Cary, NC). The frequency of each behavior relative to IPS category was analyzed. The model fitted included recording period (1, 2, or 3) and IPS category as fixed effects. Body weight was included as a covariate. Year, sire, and heifer nested within year were treated as random effects. Least squares means and SE were obtained using SAS with Tukey’s adjustment for multiple comparisons.

#### Video analyses of continuous behaviors

All other observations of heifers in the individual or pen setting considered the length of time a type of behavior was expressed within a 30-s period. Like with previous analyses, we were interested in understanding the relationships (interactions) between IPS or GPS and the expression of corresponding continuously expressed behaviors. These data were analyzed using the GLIMMIX procedure in SAS (SAS Inst. Inc., Cary, NC) fitting two separate statistical models.

First, for the individual pen setting, the length of time (s) each behavior category was expressed was evaluated using the observations collected during each recording period. A factorial model (model 1) was fitted with IPS category (1–5+), behavior category for a given ethogram (e.g., for pace, the categories stationary, walk, trot, canter, or fidget), their interaction, and recording period (1, 2, or 3) as fixed effects. Body weight was included as a covariate. Year and sire were treated as random effects. Least squares means and SE were obtained using SAS with Tukey’s adjustment for multiple comparisons.

The second model (model 2) fitted was designed to compare timed behavior categories expressed in the group pen setting. It included the fixed effect of recording period, GPS category (1-3+), behavior category, and the interaction of the GPS category and behavior category. Body weight was included as a covariate. Year and sire were treated as random effects.

## Results

### Interobserver reliability and Pearson correlation

Individual pen score had an average *K* of 0.64 and ICC of 0.92. Reliabilities of GPS were lower, with an average *K* of 0.44 and ICC of 0.77. The Pearson correlation between observer average IPS and GPS was 0.67 ± 0.04.

### Categorical behaviors

The contingency tables for behaviors observed on heifers when individually penned are provided in Table 4. In all cases, based on the chi-squared tests, observed proportions in each category of an ethogram were different than expected (*P* < 0.001). Furthermore, based on the log-linear model, goodness-of-fit improved when including the interaction of IPS category and behavior category level within each ethogram (*P* < 0.001). Heifers with an IPS less than 2.0 (IPS category of 1) had a low flight zone size, responded neutrally to a human stressor, were more likely to walk around the pen, and were calm enough in demeanor that the human felt safe to enter the pen with them. Heifers appeared less calm as IPS increased in value. As the IPS of a heifer increased to a 2.0 or 3.0 (IPS category 2), flight zone sizes increased to medium, heifers became more “flighty” than neutral, were more likely to trot or canter around the pen, and in a few cases were flighty enough that the human did not feel safe enough to enter the pen. Lastly, those heifers with IPS of 4.0 or greater (IPS category 4 or 5+) had high flight zone sizes, appeared more aggressive, expressing their fight instinct, were more likely to canter/run in the pen, and the human was much less likely to enter the pen with them.

**Table 4.** Proportion of heifers in each individual pen score category by ethogram^1^

		Individual pen score category^2^				
		1	2	3	4	5+
Ethogram	Category	*n* = 178	*n* = 77	*n* = 39	*n* = 19	*n* = 11
Flight zone size	Low	0.60	0.09	0	0	0
	Medium	0.40	0.82	0.44	0	0
	High	0	0.09	0.56	1.00	1.00
Fight or flight	Neutral	0.72	0.16	0	0	0
	Flight	0.28	0.83	0.97	0.84	0.55
	Fight	0	0.01	0.03	0.16	0.45
Fastest pace	Stationary	0.07	0	0	0	0
	Walk	0.51	0.06	0	0	0
	Trot	0.40	0.73	0.46	0.21	0
	Canter	0.02	0.21	0.54	0.79	1.00
Human entry	Safe to Enter	1.00	1.00	0.95	0.68	0.27
	Unsafe	0	0	0.05	0.32	0.73

^1^Observed and expected frequencies differed (chi-squared test *P* < 0.001).

^2^Individual pen score categories were: 1 for IPS less than 2.0; 2 for IPS equal to 2.0 but less than 3.0; 3 for IPS equal to 3.0 but less than 4.0; 4 for IPS equal to 4.0 but less than 5.0; and 5+ for IPS greater than or equal to 5.0.

Like with heifers when individually penned, observed proportions in each GPS category were different than expected based on the chi-squared tests (Table 5). Again, goodness-of-fit improved when the interaction of GPS category and the category level of an ethogram was included in the log-linear model (*P* < 0.001). Groups with average pen scores between 1.0 and 2.0 (GPS category 1) were most likely to have a low or medium flight zone size. Furthermore, their most common fastest pace was a bit quicker at a trot as compared to individuals with an IPS category of 1 who were more likely to walk. Groups with average pen scores between 2.0 and 3.0 (GPS category 2) were most likely to have a medium flight zone size and move at faster paces of a trot or canter. Lastly, groups with average pen scores greater than or equal to 3.0 (GPS category 3+) were more flighty in their movements. All groups in this category had medium to high flight zone sizes and spent their time cantering around the pen.

**Table 5.** Proportion of heifers in each group pen category by ethogram^1^

		Group pen score category^2^		
		1	2	3+
Ethogram	Category	*n* = 60	*n* = 19	*n* = 7
Flight zone size	Low	0.43	0.16	0
	Medium	0.54	0.58	0.43
	High	0.03	0.26	0.57
Fastest pace	Stationary	0	0	0
	Walk	0.15	0	0
	Trot	0.56	0.21	0
	Canter	0.29	0.79	1.00

^1^Observed and expected frequencies differed (chi-squared test *P* < 0.001).

^2^Group pen score categories were: 1 for average GPS less than 2.0; 2 for average GPS equal to 2.0 but less than 3.0; and 3+ for average GPS of greater or equal to 3.0.

### Counted behaviors

Table 6 provides mean values for each of the counted behaviors by IPS category. When comparing heifers across all recording periods, IPS category defined variation in all counted behaviors recorded (*P* < 0.05) while recording period did not (*P* > 0.24). All counted behaviors occurred more often with increasing IPS, with heifers with IPS category of 4 or 5+ consistently having the highest values. The number of times heifers head butted or hit the fence increased (*P* < 0.05) when comparing heifers with an IPS category of 2 (0.01 and 0.09 times in a 30-s window, respectively) to heifers with an IPS category of 4 (1.19 and 3.52 times in a 30-s window, respectively). Similarly, the number of times heifers flicked their tails increased from 1.87 times (IPS category 2) to 14.88 times in a 30-s window (IPS category 4). Likewise, the number of threats was highest and most likely to occur in heifers with an IPS category of 5+.

**Table 6.** Least squares means of the number of times each behavior was observed in each individual pen score category

	Individual pen score category^1^				
Behavior	1	2	3	4	5+
Escape	0.02 ± 0.06^a^	0.19 ± 0.09^a^	0.25 ± 0.13^ac^	0.87 ± 0.18^b^	0.94 ± 0.23^bc^
Head butt fence	0.01 ± 0.04^a^	0.01 ± 0.06^ab^	0.25 ± 0.08^b^	1.19 ± 0.11^c^	1.64 ± 0.15^c^
Hit fence	0.01 ± 0.10^a^	0.09 ± 0.14^a^	0.79 ± 0.19^b^	3.52 ± 0.27^c^	3.59 ± 0.35^c^
Tail flick	0.96 ± 0.38^a^	1.87 ± 0.54^a^	5.52 ± 0.77^b^	14.88 ± 1.09^c^	17.27 ± 1.42^c^
Threats	0.00 ± 0.03^a^	0.01 ± 0.04^a^	0.04 ± 0.06^a^	0.44 ± 0.08^b^	1.43 ± 0.10^c^

^1^Individual pen score categories were: 1 for IPS less than 2.0; 2 for IPS equal to 2.0 but less than 3.0; 3 for IPS equal to 3.0 but less than 4.0; 4 for IPS equal to 4.0 but less than 5.0; and 5+ for IPS greater than or equal to 5.0.

^ab^Rows with differing superscripts differ (*P* < 0.05).

### Continuous behaviors of individual pen score

For pace and direction of movement, the interaction of IPS category and behavior category defined variation (*P* < 0.01) in the time the behavior was expressed. Such was not the case for recording period (*P* > 0.98).

**Figure 3.**
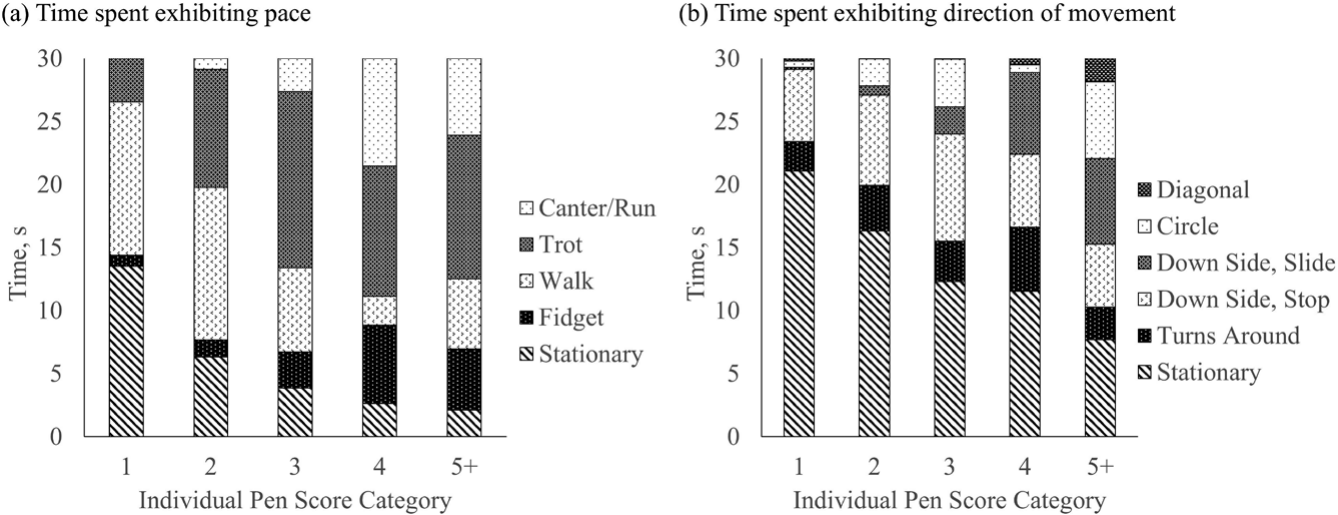
Least squares means of the time (s) heifers within each individual pen score category^1^ spent exhibiting each (a) pace and (b) direction of movement. ^1^Individual pen score categories were: 1 for IPS less than 2.0; 2 for IPS equal to 2.0 but less than 3.0; 3 for IPS equal to 3.0 but less than 4.0; 4 for IPS equal to 4.0 but less than 5.0; and 5+ for IPS greater than or equal to 5.0.

Least squares means for the amount of time spent exhibiting each pace by IPS category are shown in Figure 3a (Supplementary Table S1). Heifers with IPS category 1 spent more time standing still and/or walking than any other pace (*P* < 0.05). They also spent the most time stationary (13.52 ± 1.37 s) compared to any other IPS category. Heifers with an IPS category of 2 increased their pace to walking and trotting, with little to no running (*P* < 0.05). There was no difference in time spent trotting among higher IPS categories of 2 to 5+ (*P* > 0.05). Heifers with an IPS category of 3 spent majority of their time trotting (13.94 ± 1.12 s) compared to other paces (*P* < 0.05). While heifers in the highest IPS categories of 4 and 5+ were similar in the amount of time they expressed each pace (*P* > 0.05), they spent more time in motion (walk, trot, canter) than stationary.

Least squares means for the amount of time spent exhibiting each direction of movement by IPS category are provided in Figure 3b (Supplementary Table S2). Expectedly, heifers with higher IPS spent more time in motion compared to stationary (*P* < 0.05). Heifers appeared to move more once they reached an IPS category of 3 to 5+. Heifers in IPS category 3 spent only 2.14 ± 0.73 s sliding into the corner of the pen and 8.50 ± 0.73 s moving down the fence line and coming to a complete stop (*P* < 0.05). Comparatively, heifers with an IPS category of 4 or 5+ spent less time coming to a complete stop (5.77 ± 1.01 and 4.98 ± 1.32 s, respectively) and instead spent more time sliding into the corner of the pen (6.46 ± 1.01 and 6.82 ± 1.32 s, respectively). Heifers with an IPS category of 1–3 were potentially moving at slow enough speeds to bring themselves to a full stop compared to more excitable counterparts. Numerically, heifers with higher IPS categories of 4 or 5+ were the only heifers willing to move diagonally through the pen. This in part was because the human did not feel safe enough to enter and therefore was not present.

### Continuous behaviors of group pen score

**Figure 4.**
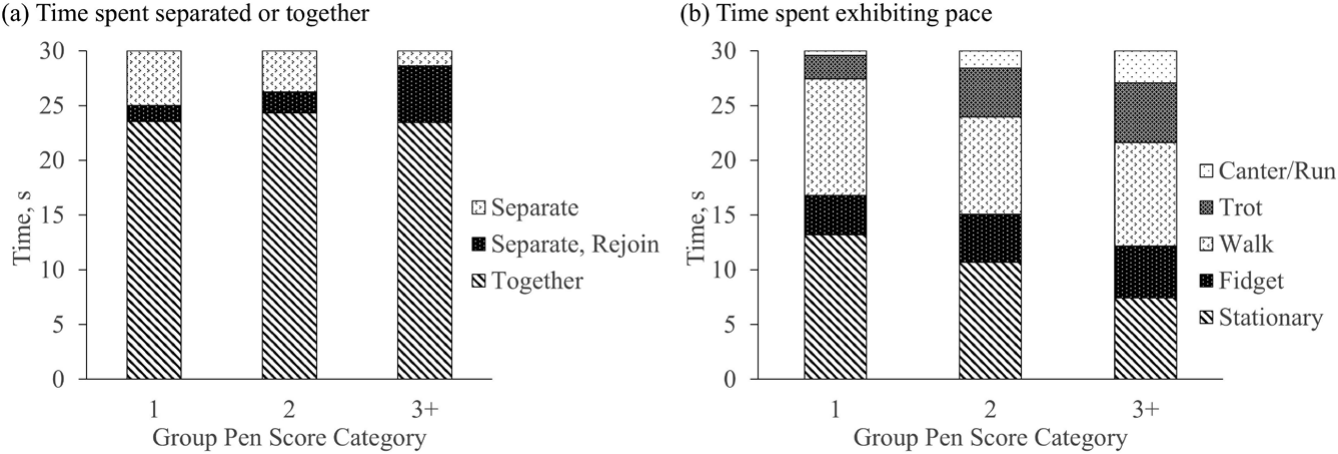
Least squares means of the time (s) heifers within each group pen score category^1^ spent (a) separated or together in a pen and (b) exhibiting each pace in a pen. ^1^Group pen score categories were: 1 for average GPS less than 2.0; 2 for average GPS equal to 2.0 but less than 3.0; and 3+ for average GPS of greater or equal to 3.0.

There was no significant interaction of GPS category and behavior category when comparing a group of heifer’s willingness to separate in the pen (*P* = 0.33). However, regardless of GPS category or recording period, heifers preferred to spend the most time together as compared to separate or to separate and rejoin (*P* < 0.05; Figure 4a, Supplementary Table S3). Although not significant, groups in GPS category 1 and 2 were more likely to remain separated (4.97 ± 0.97 and 3.76 ± 1.44 s, respectively) than those with higher average GPS (1.36 ± 2.23 s) that were more likely to be seen together (23.46 ± 2.23 s) or to rejoin when separated (5.18 ± 2.23 s).

Finally, although recording period was still not significant (*P* > 0.99), the interaction of GPS category and behavior category explained variation (*P* < 0.01) in the amount of time a heifer spent exhibiting each pace. Least squares means are provided in Figure 4b (Supplementary Table S4) by group pen score category. Again, heifers in the lowest GPS category spent the most time stationary (13.21 ± 0.75 s) compared to those with a GPS of 3+ (7.43 ± 1.32 s, *P* < 0.05). Similarly, heifers in GPS categories of 1 and 2 spent majority of their time in slower paces and the least amount of time running around the pen (*P* < 0.05).

## Discussion

A detailed analysis of the behaviors underpinning the IPS and GPS score supports their efficacy in delineating temperament when one or more beef heifers were unrestrained in a pen. Specifically, animals with higher IPS and average GPS had larger flight zone sizes and reached faster paces for longer periods of time. Furthermore, animals with lower IPS appeared more neutral to the presence of a human, moved around less often, and exhibited less behaviors such as hitting the fence and flicking their tails. Groups with higher average GPS were less willing to separate from their peers. Although positively correlated, IPS and GPS appear to describe different behavioral expressions, or responses to different levels of stress. Overall, evaluating an animal while penned individually is more straightforward than assessing them as part of a group. Combined with the fact that IPS resulted in more visually detectable, or extreme, responses to stress, it may be the more useful pen score for producers wishing to evaluate temperament in a nonrestrained animal. Lastly, interobserver reliabilities for IPS and GPS were consistently higher than published thresholds for acceptable accuracy (Landis and Koch, 1977; Bateson and Martin, 2021).

The flight zone is the minimum distance within which a person can approach an animal before it reacts or moves away (Grandin and Deesing, 2022) and is part of what defines an animal’s personal space. The size of an animal’s flight zone will slowly diminish with increased tameness or when the animal previously received frequent and gentle handling (Grandin, 2000). In this study, both IPS and GPS reflected the individual or group’s flight zone size. Most heifers assigned an IPS category of 1 had low flight zone sizes, those assigned a 2 mainly had medium flight zone sizes, while almost all heifers with a 4 or 5+ had high flight zone sizes. The way animals flee or respond to human stresses in their personal space, therefore, can be used to detect their temperament and easiness of subsequent handling (del Campo et al., 2008). Flight zones also reflect a heifer’s response to human presence. Most heifers with an IPS category of 1 or 2 remained neutral to the presence of a human, potentially because the human stayed in the middle of the pen and did not encroach their flight zone. Heifers with IPS category of 5+ were more likely to be aggressive, possibly due to a larger flight zone than the pen area occupied by the human stressor.

The relationship of flight zone size with average GPS for all four heifers was similar to IPS, with the frequency of high flight zone sizes increasing with increasing GPS. While it has been proposed that animals with similar genetics and previous handling experiences would have similar flight zone sizes (Grandin and Deesing, 2022), in this experiment, the group of four were a mix of several different IPS combinations and therefore different flight zone sizes. Overall, heifers assigned to GPS category of 1 had smaller flight zone sizes than those assigned to GPS category of 3+.

Another consistent delineation between IPS and GPS categories was the assigned fastest pace reached by the heifer(s) over the 30-s duration of the video. Heifers with lower IPS categories were more likely to only walk around the pen compared to those with IPS category of 3 to 5+, whose maximum pace was always either a trot or a canter. When pace was expressed as a continuous duration of time, heifers with lower IPS spent more total time walking and standing still compared to heifers in IPS categories of 4 or 5+ that preferred trotting and cantering around the pen. Similar patterns were present for GPS categories. Those groups assigned a GPS category of 2 moved at faster paces of a trot or canter, and heifers in the GPS category of 3+ cantered or ran at some point during the test.

The amount and type of ambulatory activity can be considered an indicator of an animal’s level of fear (Grignard et al., 2000). In a study conducted by Haley et al. (2005), calves that were prevented from nursing their dam for a short period via an anti-sucking device prior to weaning spent 78.9% less time walking and 24.1% more time resting than control calves weaned by abrupt separation. Furthermore, the rate at which animals move has been used to detect temperament with methods like flight speed and EV (del Campo et al., 2008; Parham et al., 2021). While these methods pertain directly to how an animal leaves a chute, the concept can be applied to other nonrestrained measures of temperament where the rate or pace at which an animal moves is indicative of their level of stress. There is little research, however, published specific to the pace of an animal and stress. Mazurek et al. (2011) observed that after seven replicates of handling in an open alley (30 m × 6 m), groups of heifers (*n* = 5) adapted to test conditions and slowed their pace from running to just walking from one end of the alley to the other. Similarly, Parham et al. (2019b, 2022) reported that cattle acclimated to repeated calm handling in the chute and in an individual pen setting. Grignard et al. (2000) measured when calves began to stand still, walk, or run in an open pen (3.5 m × 5 m) and found they spent little time running regardless of whether they were alone (0.06 s) or in the presence of a motionless human (0.18 s), suggesting the overall temperament of their calves were docile. Nevertheless, calves were significantly less still when the human was present compared to when they were absent (Grignard et al., 2000). There was a strong relationship between pace, whether evaluated categorically or continuously, and IPS and GPS in heifers observed in the current study. However, more research is needed to confirm this extrapolation of the relationship of pace in a nonrestrained setting to temperament.

Heifers assigned a higher IPS also remained in motion for longer periods. In addition to the overall pace, two other behaviors helped delineate temperaments. First was whether the heifers continuously circled the pen, as that occurred more often in more temperamental heifers. Second, was the heifers’ self-control when moving down the side of the fence. Heifers with lower IPS categories had enough control to bring themselves to a complete stop before sliding and/or hitting the fence while heifers with an IPS category of 4 or 5+ had too much momentum to do so, and, therefore, slid into the corner at times hitting the fence.

Fearful or excitable responses can be expressed by animals in any novel situation and can manifest itself in a variety of ways such as agitated movements, attempts to escape, vocalization, and changes in head and tail positions (Haskell et al., 2014). In this study, when assessing an animal’s IPS, the number of times a heifer attempted to escape, head butt or hit the fence, flicked their tail, or threatened the human in the center of the pen increased with increasing IPS, and may therefore provide more objective methods for delineating increased agitation. In other studies, these individual behaviors were summarized by entering counts into a computer software package that calculated an overall “docility score” (Le Neindre et al., 1995; Grignard et al., 2000, 2001). Grignard et al. (2001) found heifers that displayed threats or attacked the human in the pen environment had lower (worse) docility scores than those observed for all animals.

The increased reactiveness of heifers with increasing IPS category can potentially be characterized as a “fight or flight” response, defined as a major process by which an organism responds to stress or danger (Cannon, 1925). Initiated by the sympathetic–adrenal–medullary axis, the “fight or flight” response includes an integrated behavioral response to an acute stressor in conjunction with a metabolic response in the form of increased cortisol and glucose in the bloodstream (Chen et al., 2015). The correlation of IPS with serum cortisol and glucose concentrations for animals in this same study was moderately positive [*r* = 0.28; Parham et al. (2022)]. A similar relationship between cortisol and pen score (*r* = 0.29) was observed by Curley Jr et al. (2006). Animals with increased pen score are potentially experiencing or responding with more stress, thereby initiating a “fight or flight” response. In many cases, the heifer chose “flight” over “fight”, with increased pace and amount of movement around the pen in an attempt to find an escape. There were few times in this study (*n* = 16) where a heifer exhibited more of a “fight” response where the human stressor chose not to enter the pen, all of which occurred when a heifer was assigned an IPS of 3 or greater.

Since the descriptions of scores assigned by the IPS and GPS ethograms were similar, unsurprisingly the correlation between the two scores was moderate in size and positive. However, since that correlation was significantly less than one, an animal’s stress response in these two settings somewhat differed. Behaviors and trends more clearly observed when measured in an individual pen setting became diluted when heifers were observed in groups of four, potentially because of lower interobserver reliability. One potential cause of this reduction in GPS reliability could be the order in which heifers were evaluated. Based on their own choice, the three observers watched one heifer, assigned their score, and then moved to the next. By evaluating the heifers in different orders, they may have observed different expressions of behavior leading to slightly different scores being assigned to the same animal.

In addition to starting with a lower overall GPS compared to IPS (Parham et al., 2022), there was more variation in individual animal flight zone sizes within a single GPS category. The heifers also moved around the pen at a faster pace. This could be because individual cattle behavior is influenced by that of the group as a whole (Mazurek et al., 2011). This is not because particular individuals affected the group, but rather the effect of the number of animals in the group and the additional protection it offered (Mazurek et al., 2011). This protection, or antipredator behavior, has associated costs and benefits, which have been studied extensively in wild animal populations. Still, these behaviors have been maintained in domesticated species (Estevez et al., 2007). This drive to remain as part of the group was reflected in the time animals within a group spent together or in attempting to rejoin each other after being separated. The need to remain with the group could cause heifers with lower IPS to move faster in a group setting for longer periods.

Secondly, while the overall group pen size was larger (12 × 8 m) than the individual pen (12 × 6 m), there was less available space in the group pen per animal. Therefore, while still a nonrestrained score of temperament, the heifers had more restraint in their environment compared to the individual pen setting. This potential limitation to their expression of behaviors could be the reason for higher behavioral measures of pace and increased variation in fight zone sizes aligning with lower overall GPS.

Another explanation for the diluted GPS assessments, compared to IPS, could be the additional acute stress of reshuffling or comingling of different groups of heifers each time they were observed (Chen et al., 2015). However, heifers in this study were housed together before, during, and after each collection day; therefore, none would be considered a “novel” animal.

## Conclusion

The ability of an animal to display their full repertoire of behaviors in response to stress impacts the effectiveness of an ethogram. In being a nonrestrained test, evaluating an animal penned individually or in a group allows them the freedom to move and behave as they choose within the confines of the pen. Differences in flight zone will impact how stressed an animal appears when secluded in a pen with a human, and the behaviors they express in trying to escape that threat. Removing restraint therefore allows for a more comprehensive evaluation of the temperament of an animal. Most importantly, when explicit behaviors described within the pen score ethograms were categorized and quantified, they consistently aligned with a designated score. In conclusion, although it takes more time to assess, pen score, especially IPS, is a valuable and relevant summary of the repertoire of behaviors associated with temperament in cattle.

## Supplementary Material

skaf026_suppl_Supplementary_Tables

## Abbreviations

CS: chute score
ES: exit score
EV: exit velocity
GPS: group pen score
ICC: intraclass correlation
IPS: individual pen score
*K*: Fleiss’ kappa coefficient.
