# Supplementary material for: Validating the use of pen scores to capture behaviors expressed by cattle unrestrained in a pen
Source: J Anim Sci. 2025 Feb 5;103:skaf026. doi: 10.1093/jas/skaf026 (PMC11912831; doi:10.1093/jas/skaf026)
Supplement: skaf026_suppl_Supplementary_Tables [file skaf026_suppl_supplementary_tables.docx]

Table S1. Least squares means of the time (s) heifers within each individual pen score category spent exhibiting each pace.

|  | **Pace** | | | | |  |
| --- | --- | --- | --- | --- | --- | --- |
| **IPS Category^1^** | Stationary | Fidget | Walk | Trot | Canter/Run | SEM |
| 1 | 13.52^a,x^ | 0.87^b,x^ | 12.17^a,x^ | 3.44^b,x^ | 0.00^b,x^ | 0.73 |
| 2 | 6.32^a,y^ | 1.34^b,x^ | 12.12^ac,x^ | 9.35^ac,y^ | 0.87^b,x^ | 0.89 |
| 3 | 3.87^a,y^ | 2.84^a,x^ | 6.71^a,y^ | 13.94^b,y^ | 2.64^a,x^ | 1.12 |
| 4 | 2.62^a,y^ | 6.23^a,x^ | 2.29^a,y^ | 10.32^a,y^ | 8.54^a,x^ | 1.48 |
| 5+ | 2.10^a,y^ | 4.87^a,x^ | 5.53^a,xy^ | 11.41^a,y^ | 6.09^a,x^ | 1.90 |

^1^ Individual Pen Score categories were: 1 for IPS less than 2.0; 2 for IPS equal to 2.0 but less than 3.0; 3 for IPS equal to 3.0 but less than 4.0; 4 for IPS equal to 4.0 but less than 5.0; and 5+ for IPS greater than or equal to 5.0

^a-c^ Rows with different superscripts differ (*P* < 0.05)

^x-z^ Columns with different superscripts differ (*P* < 0.05)

Table S2. Least squares means of the time (s) heifers within each individual pen score category spent exhibiting each direction of movement.

|  | | **Direction of Movement** | | | | | |  |
| --- | --- | --- | --- | --- | --- | --- | --- | --- |
| **IPS Category^1^** | Stationary | | Diagonal | Circle | Down Side, Slide | Down Side, Stop | Turns Around | SEM |
| 1 | 21.08^a,x^ | | 0.21^b,x^ | 0.50^bc,x^ | 0.15^b,x^ | 5.71^e,x^ | 2.35^cd,x^ | 0.37 |
| 2 | 16.33^a,y^ | | 0.02^b,x^ | 2.17^bc,xy^ | 0.73^bc,x^ | 7.15^d,x^ | 3.60^c,x^ | 0.53 |
| 3 | 12.31^a,z^ | | 0.05^b,x^ | 3.80^b,y^ | 2.14^b,xy^ | 8.50^a,x^ | 3.20^b,x^ | 0.73 |
| 4 | 11.53^a,z^ | | 0.50^b,x^ | 0.64^b,xy^ | 6.46^ac,y^ | 5.77^bc,x^ | 5.10^bc,x^ | 1.01 |
| 5+ | 7.70^a,z^ | | 1.85^a,x^ | 6.07^a,y^ | 6.82^a,y^ | 4.98^a,x^ | 2.58^a,x^ | 1.32 |

^1^ Individual Pen Score categories were: 1 for IPS less than 2.0; 2 for IPS equal to 2.0 but less than 3.0; 3 for IPS equal to 3.0 but less than 4.0; 4 for IPS equal to 4.0 but less than 5.0; and 5+ for IPS greater than or equal to 5.0

^a-c^ Rows with different superscripts differ (*P* < 0.05)

^x-z^ Columns with different superscripts differ (*P* < 0.05)

Table S3. Least squares means of the time (s) heifers within each group pen score category spent separated or together in a pen.

|  | **Willingness to Separate** | | |  |
| --- | --- | --- | --- | --- |
| **GPS Category^1^** | Separate^a^ | Separate, Rejoin^a^ | Together^b^ | SEM |
| 1 | 4.97 | 1.47 | 23.56 | 0.97 |
| 2 | 3.76 | 1.92 | 24.32 | 1.44 |
| 3+ | 1.36 | 5.18 | 23.46 | 2.23 |

^1^ Group Pen Score Categories were: 1 for average GPS less than 2.0; 2 for average GPS equal to 2.0 but less than 3.0; and 3+ for average GPS of greater or equal to 3.0

^ab^ The interaction of GPS category and behavior was not significant (*P* > 0.05), while behavior was (*P* < 0.05).

Table S4. Least squares means for the amount of time (s) heifers within each group pen score category spent exhibiting each pace.

|  | **Pace** | | | | |  |
| --- | --- | --- | --- | --- | --- | --- |
| **GPS Category^1^** | Stationary | Fidget | Walk | Trot | Canter/Run | SEM |
| 1 | 13.21^a,x^ | 3.58^b,x^ | 10.65^a,x^ | 2.12^b,x^ | 0.44^b,x^ | 0.75 |
| 2 | 10.68^a,xy^ | 4.39^bc,x^ | 8.89^ab,x^ | 4.46^bc,x^ | 1.58^c,x^ | 0.93 |
| 3+ | 7.43^a,y^ | 4.75^a,x^ | 9.43^a,x^ | 5.45^a,x^ | 2.94^a,x^ | 1.32 |

^1^ Group Pen Score Categories were: 1 for average GPS less than 2.0; 2 for average GPS equal to 2.0 but less than 3.0; and 3+ for average GPS of greater or equal to 3.0

^a-c^ Rows with different superscripts differ (*P* < 0.05)

^x-z^ Columns with different superscripts differ (*P* < 0.05)
